# Synergetic Fermentation of Glucose and Glycerol for High-Yield N-Acetylglucosamine Production in *Escherichia coli*

**DOI:** 10.3390/ijms23020773

**Published:** 2022-01-11

**Authors:** Kaikai Wang, Xiaolu Wang, Huiying Luo, Yaru Wang, Yuan Wang, Tao Tu, Xing Qin, Yingguo Bai, Huoqing Huang, Bin Yao, Xiaoyun Su, Jie Zhang

**Affiliations:** State Key Laboratory of Animal Nutrition, Institute of Animal Science, Chinese Academy of Agricultural Sciences, Beijing 100193, China; kkw_2012@163.com (K.W.); wangxiaolu@caas.cn (X.W.); luohuiying@caas.cn (H.L.); wangyaru@caas.cn (Y.W.); wangyuan08@caas.cn (Y.W.); tutao@caas.cn (T.T.); qinxing@caas.cn (X.Q.); baiyingguo@caas.cn (Y.B.); huanghuoqing@caas.cn (H.H.); binyao@caas.cn (B.Y.)

**Keywords:** *Escherichia coli*, metabolic engineering, fructose-6-phosphate accumulation, synergetic carbon fermentation, N-acetylglucosamine

## Abstract

N-acetylglucosamine (GlcNAc) is an amino sugar that has been widely used in the nutraceutical and pharmaceutical industries. Recently, microbial production of GlcNAc has been developed. One major challenge for efficient biosynthesis of GlcNAc is to achieve appropriate carbon flux distribution between growth and production. Here, a synergistic substrate co-utilization strategy was used to address this challenge. Specifically, glycerol was utilized to support cell growth and generate glutamine and acetyl-CoA, which are amino and acetyl donors, respectively, for GlcNAc biosynthesis, while glucose was retained for GlcNAc production. Thanks to deletion of the 6-phosphofructokinase (PfkA and PfkB) and glucose-6-phosphate dehydrogenase (ZWF) genes, the main glucose catabolism pathways of *Escherichia coli* were blocked. The resultant mutant showed a severe defect in glucose consumption. Then, the GlcNAc production module containing glucosamine-6-phosphate synthase (GlmS*), glucosamine-6-phosphate N-acetyltransferase (GNA1*) and GlcNAc-6-phosphate phosphatase (YqaB) expression cassettes was introduced into the mutant, to drive the carbon flux from glucose to GlcNAc. Furthermore, co-utilization of glucose and glycerol was achieved by overexpression of glycerol kinase (GlpK) gene. Using the optimized fermentation medium, the final strain produced GlcNAc with a high stoichiometric yield of 0.64 mol/mol glucose. This study offers a promising strategy to address the challenge of distributing carbon flux in GlcNAc production.

## 1. Introduction

N-acetylglucosamine (GlcNAc) is the monomer unit of chitin, which is the second most abundant polysaccharide on Earth and can be commonly found in crustaceans, fungi and insects [1]. It is also a basic component of various heterologous biopolymers, such as hyaluronic acid and chondroitin sulfate, which play important roles in cartilage and joint health [2,3]. Furthermore, the GlcNAc molecule can be frequently observed in glycoproteins, mammalian growth factors and hormones, which are directly involved in a broad range of physiological functions [4]. Due to its unique characteristics, GlcNAc and its derivatives have received extensive attention for their commercial applications in the healthcare, cosmetics and pharmaceutical industries [5].

**Figure 1:**
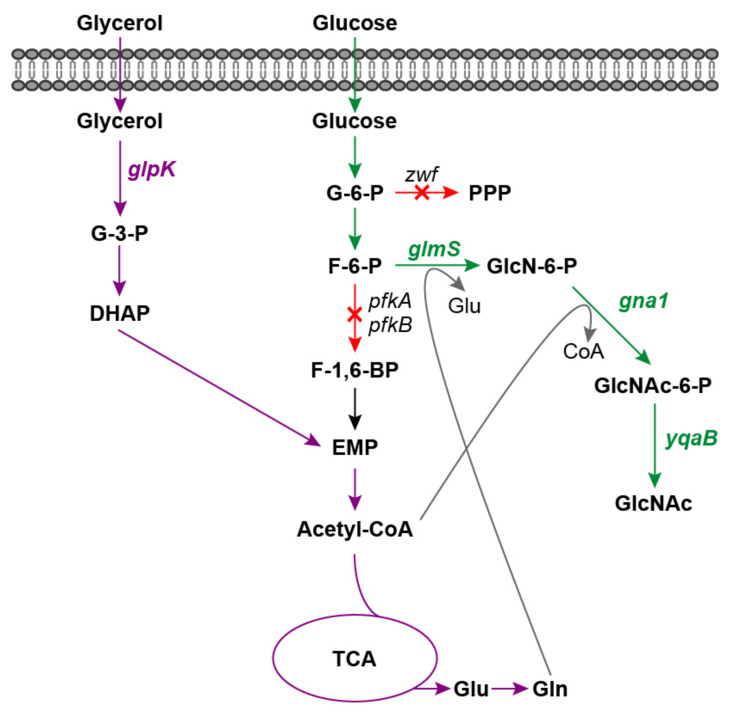
Schematic diagram of GlcNAc production via synergistic carbon co-utilization mechanism. Red arrows and crosses indicate gene deletions; green arrows indicate the GlcNAc biosynthesis pathway from glucose; purple arrows indicate glycerol utilization pathway. G-3-P, glycerol-3-phosphate; DHAP, glycerone phosphate; G-6-P, glucose-6-phosphate; F-6-P, fructose-6-phosphate; F-1,6-BP, fructose-1,6-bisphosphate; EMP, Embden-Meyerhof-Parnas pathway; TCA, tricarboxylic acid cycle; Glu, glutamic acid; Gln, glutamine; PPP, pentose phosphate pathway; GlcN-6-P, glucosamine-6-phosphate; GlcNAc, N-acetylglucosamine; *glpK*, glycerol kinase gene; *zwf*, glucose-6-phosphate dehydrogenase gene; *glmS*, glucosamine-6-phosphate synthase gene; *gna1*, glucosamine-6-phosphate N-acetyltransferase gene; *yqaB*, GlcNAc-6-phosphate phosphatase gene.

Traditionally, GlcNAc is produced through chemical and enzymatic hydrolysis of crustacean shells [6,7]. However, there are several drawbacks to these extraction processes, such as the limitation of raw material supply and severe environmental pollution. Moreover, GlcNAc is difficult to extract from crab and shrimp shells without allergenic risk for individuals who suffer from shellfish allergies. In recent years, microbial production of GlcNAc has drawn tremendous attention, as it is a promising alternative to the production of non-shellfish-derived GlcNAc in a low-cost and environmentally compatible manner [8,9,10]. Several microorganism species have been evaluated for GlcNAc production, including *Escherichia coli* [11], *Bacillus subtilis* [12], *Saccharomyces cerevisiae* [13], *Lactobacillus plantarum* [14] and *Corynebacterium glutamicum* [15]. The biosynthesis pathway of GlcNAc from the precursor fructose-6-phosphate (F-6-P) involves three crucial enzymes, glucosamine-6-phosphate synthase (GlmS), glucosamine-6-phosphate *N*-acetyltransferase (GNA1) and GlcNAc-6-phosphate phosphatase (Figure 1). Various metabolic engineering strategies have been applied to improve GlcNAc production and the current efforts are focused largely on enhancing the GlcNAc biosynthesis pathway through key enzyme screening and overexpression, deleting by-product biosynthetic pathways, blocking catabolism of intracellular GlcNAc and engineering transcription factors [15,16,17]. The key precursor for GlcNAc biosynthesis is F-6-P, which is also an essential intermediate for the Embden-Meyerhof-Parnas pathway (EMP). Furthermore, sufficient supplies of glutamine and acetyl-CoA, which act as amino and acetyl donors, are also important for GlcNAc production (Figure 1). Therefore, modulation of the balance between cell growth and GlcNAc biosynthesis is crucial for high-level GlcNAc production. However, there are relatively few studies focusing on this strategy, which may limit the further improvement of microbial production of GlcNAc.

Cell metabolism can be rationally divided into growth and production modules by using mixed substrates with direct access to multiple pathways. Based on this strategy, production enhancements have been widely reported. For example, in myo-inositol fermentation, a creative strategy has been exploited for efficient inositol production (reaching as high as 106.3 g/L) by synergetic utilization of glucose and glycerol as carbon sources [18]. Additionally, the productivity of lycopene was significantly improved in the fed-batch culture of glycerol supplemented with glucose and arabinose, which was 11.7-fold higher than that without auxiliary carbon sources [19]. Furthermore, it was reported that by controlled cofeeding of ATP and NADPH generators, such as glucose and gluconate, CO_2_ reduction and CO_2_-derived lipid production were dramatically accelerated compared to the CO_2_-only control [20]. Thus, the synergistic substrate cofeeding strategy represents a good option to modulate carbon flux distribution in GlcNAc biosynthesis.

In this study, our aim was to achieve high GlcNAc production by modulation of cell growth and GlcNAc biosynthesis using the synergistic substrate cofeeding strategy with glucose and glycerol. First, the glucose utilization pathways of *E. coli*, including EMP and the pentose phosphate pathway (PPP), were blocked by deleting the 6-phosphofructokinase genes (*pfkA* and *pfkB*) and glucose-6-phosphate dehydrogenase gene (*zwf*). Second, the glycerol consumption pathway and the GlcNAc biosynthesis pathway were enhanced. Consequently, glucose would be conserved for GlcNAc production while glycerol would be used to support cell growth and supply glutamine and acetyl-CoA for GlcNAc biosynthesis. Finally, the fermentation medium was optimized and GlcNAc production reached 2.62 g/L with a stoichiometric yield of 0.64 mol GlcNAc/mol glucose in shake flask fermentation. The results from this study provide valuable guidance and an essential reference for achieving rational distribution of carbon flux for the production of other value-added biochemicals.

## 2. Results

### 2.1. Construction and Characterization of an E. coli Platform Strain with High F-6-P Supply

To achieve a high yield of GlcNAc from glucose, carbon flux distribution at principal branch points (glucose-6-phosphate (G-6-P) and F-6-P) in the central metabolic network of *E. coli* must be significantly modified from that observed during balanced growth, so that the GlcNAc precursor F-6-P can be synthesized in the optimal stoichiometric ratio. For *E. coli* strains, G-6-P could be driven toward PPP through ZWF, while F-6-P was mainly broken down to pyruvate in EMP via PFK encoded by the *pfkA* and *pfkB* genes (Figure 1). Therefore, the *zwf*, *pfkA* and *pfkB* genes were successively knocked out in *E. coli* MG1655(DE3) using the CRISPR-Cas9 system to block PPP and EMP [21], generating mutants MG1655(DE3)∆*pfkA*, MG1655(DE3)∆*pfkB*, MG1655(DE3)∆*pfkA*∆*pfkB*, MG1655(DE3)∆*zwf* and MG1655(DE3)∆*pfkA*∆*pfkB*∆*zwf*.

**Figure 2:**
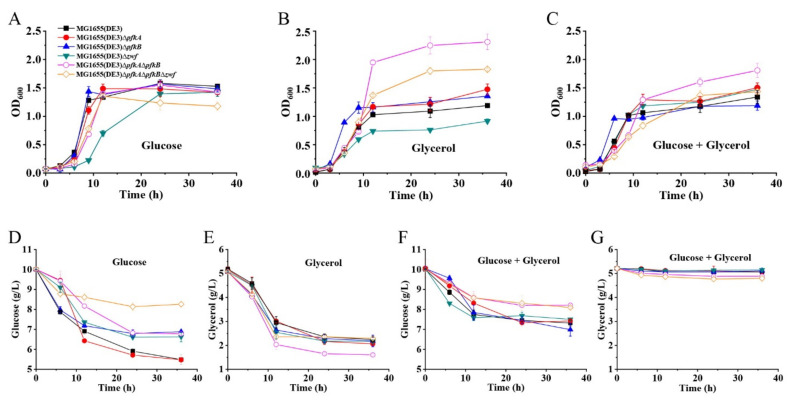
Characterization of mutants MG1655(DE3)∆*pfkA*, MG1655(DE3)∆*pfkB*, MG1655(DE3)∆*zwf*, MG1655(DE3)∆*pfkA*∆*pfkB* and MG1655(DE3)∆*pfkA*∆*pfkB*∆*zwf*. Growth profiles of various strains cultivated in (**A**) M9s+glucose medium, (**B**) M9s+glycerol medium, (**C**) M9s+glucose+glycerol medium; (**D**) glucose consumption of various strains cultivated in M9s+glucose medium; (**E**) glycerol consumption of various strains cultivated in M9s+glycerol medium; (**F**) glucose consumption of various strains cultivated in M9s+glucose+glycerol medium; (**G**) glycerol consumption of various strains cultivated in M9s+glucose+glycerol medium. Mean values are based on three independent replicates.

The growth profiles of the metabolically engineered strains and the *E. coli* MG1655(DE3) wild-type strain were then compared on various media, including an M9s medium with different carbon sources (glucose, glycerol or glucose+glycerol) (Figure 2). Glucose and glycerol, which enter the EMP upstream or downstream of F-6-P, were chosen. As shown in Figure 2A–C, the growth of mutants with a single deletion of the *pfkA* or *pfkB* gene (MG1655(DE3)∆*pfkA* or MG1655(DE3)∆*pfkB*) was almost unaffected compared to the wild-type strain under all tested culture conditions. However, deletion of the *zwf* gene (MG1655(DE3)∆*zwf*) led to a slight decrease of growth rate, which might be due to the inefficient supply of NADPH (Figure 2A,B). Double-deletion mutant (MG1655(DE3)∆*pfkA*∆*pfkB*) and triple-deletion mutant (MG1655(DE3)∆*pfkA*∆*pfkB*∆*zwf*) showed increased maximum OD600 on a glycerol medium (Figure 2B). Surprisingly, the mutant MG1655(DE3)∆*pfkA*∆*pfkB*∆*zwf* with a blocked PPP and EMP could grow well under the culture condition where glucose was used as the sole carbon source (Figure 2A).

The glucose and glycerol consumptions of different MG1655(DE3) mutants on various media are given in Figure 2D–G. Although the triple-deletion mutant MG1655(DE3)∆*pfkA*∆*pfkB*∆*zwf* could grow on a glucose medium, the glucose consumption was significantly lower than those of the wild-type strain and the single- or double-deletion mutants (Figure 2D), which would result in the intracellular accumulation of F-6-P. As expected, the presence of glucose inhibited the consumption of glycerol for the MG1655(DE3) wild-type strain and three single-deletion mutants via carbon catabolite repression. In contrast, the carbon catabolite repression was mildly alleviated for mutants MG1655(DE3)∆*pfkA*∆*pfkB* and MG1655(DE3)∆*pfkA*∆*pfkB*∆*zwf* with 0.33 and 0.45 g/L glycerol consumed, respectively, when glucose and glycerol were used in a mixed carbon source (Figure 2F,G). This phenomenon coincides with the result reported by Shiue and co-workers that significantly reduced glucose transportation and utilization in the cell would result in the alleviation of carbon catabolite repression [22]. However, only a small amount of glycerol was consumed in our mixed-carbon-source fermentation. This might be because mutants MG1655(DE3)∆*pfkA*∆*pfkB* and MG1655(DE3)∆*pfkA*∆*pfkB*∆*zwf* still have a weak glucose-utilization ability.

**Figure 3:**
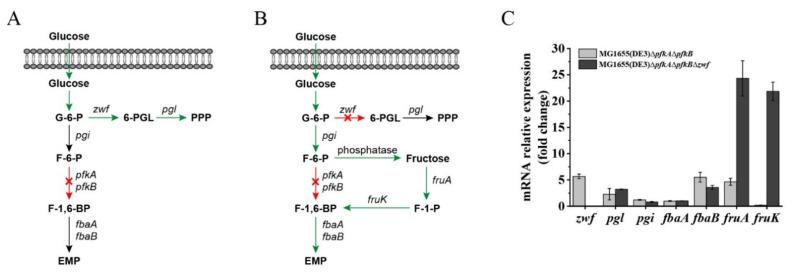
Gene expression and glucose utilization patterns of MG1655(DE3)∆*pfkA*∆*pfkB* and MG1655(DE3)∆*pfkA*∆*pfkB*∆*zwf* cultivated in M9s+glucose medium. Schematic diagrams of proposed glucose utilization patterns of (**A**) MG1655(DE3)∆*pfkA*∆*pfkB* and (**B**) MG1655(DE3)∆*pfkA*∆*pfkB*∆*zwf* (F-1-P bypass); (**C**) qPCR results showing the mRNA expression of genes related to G-6-P, F-6-P and F-1,6-BP metabolism in MG1655(DE3)∆*pfkA*∆*pfkB* and MG1655(DE3)∆*pfkA*∆*pfkB*∆*zwf*. 6-PGL, 6-phosphogluconolactone; *pgl*, 6-phosphogluconolactonase gene; F-1-P, fructose-1-phosphate; *pgi*, glucose-6-phosphate isomerase gene; *fruA*, fructose-specific PTS multiphosphoryl transferase gene; *fruK*, 1-phosphofructokinase; *fbaA*, fructose-bisphosphate aldolase gene (class 2); *fbaB*, fructose-bisphosphate aldolase gene (class 1).

Recently, the construction of an *E. coli* ∆*pfkA*∆*pfkB*∆*zwf* triple-deletion mutant has been reported by several groups, and the published data showed that this strain has a severe growth defect when glucose is the sole carbon source [23,24,25,26]. However, our results demonstrated that mutant MG1655(DE3)∆*pfkA*∆*pfkB*∆*zwf* could still consume glucose through an unknown pathway. Font et al. reported that the strain *E. coli* LJ110∆*pfkA*∆*pfkB*∆*zwf* showed no growth on a glucose medium but could form small colonies on fructose agar plates [26]. Because the intracellular F-6-P could accumulate after deletion of the *pfkA*, *pfkB* and *zwf* genes, we assumed that F-6-P might enter the lower glycolytic trunk via an F-1-P bypass (Figure 3B), whereby (1) F-6-P is dephosphorylated by phosphatase to form fructose and (2) fructose is then subsequently converted to fructose-1,6-bisphosphate (F-1,6-BP) by enzyme II^fru^ of the phosphoenolpyruvate (PEP)-dependent phosphotransferase system (PTS) and 1-phosphofructokinase encoded by *fruA* and *fruK*, respectively. To verify our hypothesis, RT-qPCR was performed to analyze the expression of genes closely related to G-6-P, F-6-P and F-1,6-BP metabolism (Figure 3C). Dephosphorylation is usually mediated by phosphatases; however, these kinds of enzymes typically act on multiple substrates [27,28]. The specific phosphatase responsible for the conversion of F-6-P to fructose is unknown. Therefore, the expression of F-6-P phosphatase was not detected. The qPCR results showed that the expressions of the *zwf* and *pgl* (encoding 6-phosphogluconolactonase) genes were upregulated in MG1655(DE3)∆*pfkA*∆*pfkB*, while the expression of *pgi* (encoding G-6-P isomerase) was almost unchanged, indicating that more carbon flux was driven to the PPP after the EMP was blocked (Figure 3A,C). When both the EMP and PPP were blocked, the expression of the *fruA*, *fruK* and *fbaB* genes was significantly enhanced in MG1655(DE3)∆*pfkA*∆*pfkB*∆*zwf* (24.3-, 21.9- and 3.6-fold increase, respectively), demonstrating that the accumulated F-6-P could be channeled through an F-1-P bypass to the downstream EMP (Figure 3B,C).

### 2.2. GlcNAc Production by Synergetic Utilization of Glucose and Glycerol

**Figure 4:**
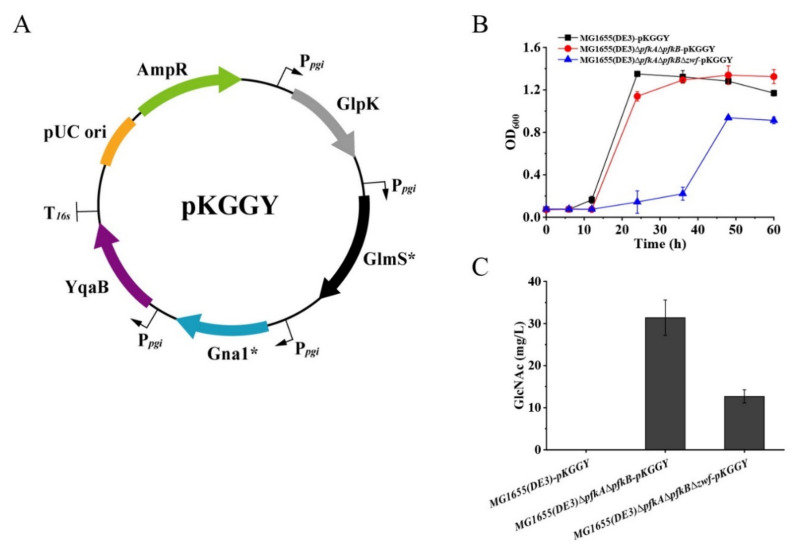
Batch fermentation profiles of MG1655(DE3)-pKGGY, MG1655(DE3)∆*pfkA*∆*pfkB-*pKGGY and MG1655(DE3)∆*pfkA*∆*pfkB*∆*zwf-*pKGGY grown in M9s+glucose+glycerol medium. (**A**) Plasmid pKGGY was constructed for the overexpression of *glpK*, glmS*, gna1* and *yqaB* genes; (**B**) cell growth; (**C**) GlcNAc production.

*E. coli* has a native biosynthetic pathway of GlcNAc, which is used for the synthesis of peptidoglycan and lipopolysaccharide, the essential components of the cell wall of the *E. coli* strain. However, the synthetic pathway for GlcNAc in *E. coli* is tightly regulated. Therefore, the glmS* gene from *E. coli* [29], gna1* gene from *Caenorhabditis elegans* [30] and *yqaB* gene (GlcNAc-6-phosphate phosphatase) from *E. coli* [17] were selected for overexpression under the control of the native *pgi* promoter to convert F-6-P to GlcNAc. Although carbon catabolite repression was alleviated for mutants MG1655(DE3)∆*pfkA*∆*pfkB* and MG1655(DE3)∆*pfkA*∆*pfkB*∆*zwf*, glycerol consumption was still restricted in the presence of glucose (Figure 2F,G). To achieve high GlcNAc production by synergetic fermentation of glucose and glycerol, the glycerol utilization pathway must be further enhanced. Glycerol kinase (GlpK) catalyzes the phosphorylation of glycerol to yield glycerol-3-phosphate (G-3-P), which is the rate-limiting step in glycerol utilization. Thus, the *glpK* gene from *Pichia pastoris* GS115 (a well-known glycerol utilization organism) was chosen for overexpression to enhance glycerol utilization in the presence of glucose. Plasmid pKGGY harboring *glpK*, glmS*, gna1* and *yqaB* expression cassettes was then constructed based on the pEASY-T3 mother vector (Figure 4A) and introduced into the MG1655(DE3) wild-type strain, MG1655(DE3)∆*pfkA*∆*pfkB* and MG1655(DE3)∆*pfkA*∆*pfkB*∆*zwf*, generating mutants MG1655(DE3)-pKGGY, MG1655(DE3)∆*pfkA*∆*pfkB-*pKGGY and MG1655(DE3)∆*pfkA*∆*pfkB*∆*zwf-*pKGGY, respectively.

Batch fermentation of mutants to produce GlcNAc was carried out in shake flask at 37 °C. M9s supplemented with 10 g/L glucose and 5 g/L glycerol was used as the fermentation medium. The results demonstrated that the growth of mutant MG1655(DE3)∆*pfkA*∆*pfkB*∆*zwf-*pKGGY was significantly decreased compared to those of MG1655(DE3)-pKGGY and MG1655(DE3)∆*pfkA*∆*pfkB-*pKGGY for unknown reason (Figure 4B). No GlcNAc was detected in the fermentation broth of MG1655(DE3)-pKGGY, while mutants MG1655(DE3)∆*pfkA*∆*pfkB-*pKGGY and MG1655(DE3)∆*pfkA*∆*pfkB*∆*zwf-*pKGGY produced 31.4 and 12.7 mg/L GlcNAc, respectively, after 36 h of fermentation (Figure 4C), indicating that rational carbon flux distribution is important for GlcNAc production.

### 2.3. Medium Optimization for Enhanced Growth of MG1655(DE3)∆pfkA∆pfkB∆zwf-pKGGY

**Figure 5:**
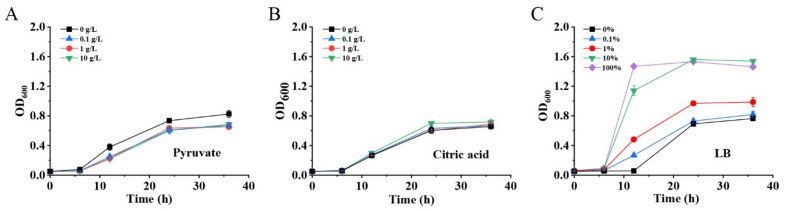
Effects of pyruvate (**A**), citric acid (**B**) and LB (**C**) addition on cell growth of MG1655(DE3)∆*pfkA*∆*pfkB*∆*zwf-*pKGGY.

It is well known that cell biomass production during the cell growth phase is usually important to the end-product’s biosynthesis. Although GlcNAc was detected in the fermentation broth of MG1655(DE3)∆*pfkA*∆*pfkB*∆*zwf-*pKGGY, the production was still very low, mostly due to the growth restriction of this strain (Figure 4B). Two reasons may account for the growth defect of a triple-deletion mutant harboring plasmid pKGGY: (1) carbon flux distribution for cell growth is not sufficient; (2) the biosynthesis of essential nutrients is restricted. Therefore, to enhance cell growth and biomass production, various concentrations of pyruvate (EMP intermediate, providing additional carbon source for cell growth), citric acid (tricarboxylic acid (TCA) cycle intermediate, providing additional carbon source for cell growth) and LB broth (providing essential nutrients) were added into the fermentation medium (Figure 5). The results demonstrated that the addition of pyruvate and citric acid did not increase the growth rate and cell biomass of MG1655(DE3)∆*pfkA*∆*pfkB*∆*zwf-*pKGGY (Figure 5A,B), indicating that carbon flux distribution might not be the cause of the growth defect of this strain. However, the cell growth of MG1655(DE3)∆*pfkA*∆*pfkB*∆*zwf-*pKGGY was reinstalled when more than 10% LB broth was added to the medium (Figure 5C), suggesting that the growth was probably limited by some essential nutrients. Thus, the medium M9s + 10 g/L glucose + 5 g/L glycerol + 10% LB was used as the fermentation medium for the following experiments.

### 2.4. GlcNAc Production Using the Optimized Fermentation Medium

**Figure 6:**
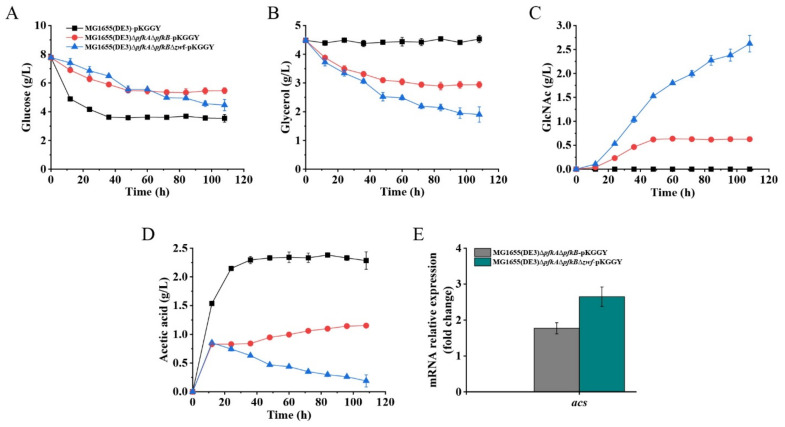
Batch fermentation profiles of MG1655(DE3)-pKGGY, MG1655(DE3)∆*pfkA*∆*pfkB-*pKGGY and MG1655(DE3)∆*pfkA*∆*pfkB*∆*zwf-*pKGGY grown in M9s+glucose+glycerol medium supplemented with 10% LB. (**A**) Glucose consumption; (**B**) glycerol consumption; (**C**) GlcNAc production; (**D**) acetic acid production; (**E**) qPCR results showing the mRNA expression of the *acs* gene.

Batch fermentation of mutants MG1655(DE3)-pKGGY, MG1655(DE3)∆*pfkA*∆*pfkB-*pKGGY and MG1655(DE3)∆*pfkA*∆*pfkB*∆*zwf-*pKGGY was performed using the optimized fermentation medium to verify its effect on GlcNAc production (Figure 6). As expected, the presence of glucose inhibited the utilization of glycerol in strain MG1655(DE3)-pKGGY via carbon catabolite repression. On the contrary, co-utilization of glucose and glycerol was apparent in mutants MG1655(DE3)∆*pfkA*∆*pfkB-*pKGGY and MG1655(DE3)∆*pfkA*∆*pfkB*∆*zwf-*pKGGY (Figure 6A,B). These results coincide with the finding that catabolite repression is alleviated in MG1655(DE3)∆*pfkA*∆*pfkB* and MG1655(DE3)∆*pfkA*∆*pfkB*∆*zwf* (Figure 2F,G) and indicate that overexpression of *glpK* from *P. pastoris* could enhance glycerol utilization. Using the optimized fermentation medium, strain MG1655(DE3)-pKGGY still could not form GlcNAc, whereas 0.63 and 2.62 g/L GlcNAc were produced by MG1655(DE3)∆*pfkA*∆*pfkB-*pKGGY and MG1655(DE3)∆*pfkA*∆*pfkB*∆*zwf-*pKGGY (Figure 6C), respectively, representing an increase of about 20- and 206-fold, respectively, compared to those using the previous fermentation medium. The stoichiometric GlcNAc yield of MG1655(DE3)∆*pfkA*∆*pfkB-*pKGGY and MG1655(DE3)∆*pfkA*∆*pfkB*∆*zwf-*pKGGY reached 0.22 and 0.64 mol/mol glucose, respectively. Other than GlcNAc, acetic acid is the major by-product of the fermentation of MG1655(DE3) mutant strains. As shown in Figure 6D, MG1655(DE3)-pKGGY generated 2.28 g/L acetic acid, while mutant MG1655(DE3)∆*pfkA*∆*pfkB-*pKGGY produced 1.15 g/L acetic acid, which decreased about 50% compared to that of MG1655(DE3)-pKGGY. Interestingly, the acetic acid production of MG1655(DE3)∆*pfkA*∆*pfkB*∆*zwf-*pKGGY reached a maximum value of 0.85 g/L at 12 h, and then this by-product was re-assimilated during the fermentation process, leaving only 0.19 g/L acetic acid at the end of fermentation. In the *E. coli* strain, acetic acid re-assimilation is usually mediated by acetyl-CoA synthetase (ACS) [31,32]. To figure out why mutants MG1655(DE3)∆*pfkA*∆*pfkB-*pKGGY and MG1655(DE3)∆*pfkA*∆*pfkB*∆*zwf-*pKGGY produced much less acetic acid than MG1655(DE3)-pKGGY, the expression of the *acs* gene was analyzed by RT-qPCR. The results showed that the *acs* expression levels of MG1655(DE3)∆*pfkA*∆*pfkB-*pKGGY and MG1655(DE3)∆*pfkA*∆*pfkB*∆*zwf-*pKGGY were indeed upregulated by 1.8- and 2.7-fold, respectively (Figure 6E). ACS catalyzes the conversion of acetic acid to acetyl-CoA, which is the acetyl donor for GlcNAc biosynthesis. Hence, acetic acid re-assimilation could be beneficial to GlcNAc production.

## 3. Discussion

Metabolic engineering aims to achieve high-yield production of value-added chemicals in engineered strains, making them economically feasible in commercial production. To achieve this goal, the target pathway is usually boosted, while competing pathways are eliminated or attenuated. However, when the competing pathways are related to the central metabolism, especially the EMP and PPP, application of this strategy becomes challenging due to their important effects on cell growth. Carbon cofeeding has been successfully used to balance growth and production metabolism, demonstrating the effectiveness of this strategy [18,19]. In this study, the carbon cofeeding strategy was successfully adopted for high-yield GlcNAc production.

The main challenge for high-level GlcNAc biosynthesis using microbial cell factory is the sufficient supply of F-6-P, which is the precursor for GlcNAc biosynthesis and the important intermediate for EMP and PPP. Disruption or attenuation of EMP and PPP could increase the yield of bioproducts derived directly from F-6-P. To improve the GlcNAc yield from glucose, a triple-deletion mutant, MG1655(DE3)∆*pfkA*∆*pfkB*∆*zwf*, was constructed with the EMP and PPP of this mutant blocked. Our results showed that this mutant could still utilize glucose, probably through an F-1-P bypass catalyzed by F-6-P phosphatase, FruA and FruK (Figure 3B). However, results from other groups showed that the triple-deletion mutant of *E. coli* (∆*pfkA*∆*pfkB*∆*zwf*) could not grow on a glucose medium [26]. This phenomenon might be caused by the existence of phage DE3 on the genome of *E. coli* MG1655, which can alter gene expression and regulation of the host. Although the triple-deletion mutant in our study could consume glucose, fermentation results revealed that most of the glucose in the fermentation broth of MG1655(DE3)∆*pfkA*∆*pfkB*∆*zwf-*pKGGY was converted to GlcNAc (0.64 mol GlcNAc/mol glucose) when glucose and glycerol were used as the mixed carbon source (Figure 6A,C), indicating that blocking the EMP and PPP can favor GlcNAc production. In addition, repression of glycerol utilization by glucose was alleviated in mutant MG1655(DE3)∆*pfkA*∆*pfkB*∆*zwf* (Figure 2F,G), which facilitated the co-utilization of glucose and glycerol for GlcNAc production and cell growth. Moreover, the glycerol utilization was further enhanced via overexpression of *glpK* gene from *P. pastoris*.

A growth defect was observed for mutant MG1655(DE3)∆*pfkA*∆*pfkB*∆*zwf-*pKGGY, which badly influenced GlcNAc production. The GlcNAc production of MG1655(DE3)∆*pfkA*∆*pfkB*∆*zwf-*pKGGY was much lower than that of MG1655(DE3)∆*pfkA*∆*pfkB*-pKGGY when M9s supplemented with glucose and glycerol was used as the fermentation medium. Yet, the addition of LB broth (>10% *v/v*) could dramatically increase the triple-deletion mutant’s growth and GlcNAc production (over 200-fold rise) (Figure 5C). Finally, the GlcNAc production of MG1655(DE3)∆*pfkA*∆*pfkB*∆*zwf-*pKGGY reached 2.62 g/L using the optimized medium, representing an increase of about 3.2-fold compared to that of mutant MG1655(DE3)∆*pfkA*∆*pfkB*-pKGGY. These results implied that the biosynthesis of some essential nutrients was limited due to the expression of genes related to GlcNAc production and glycerol utilization. Further investigations are required to determine the physiological causes of the observed phenomena.

Industrial fermentations of *E. coli* strains are usually plagued by unproductive conversion of glucose to acetate, which leads to low product yields and inhibition of cell growth [33,34]. In this study, most of the acetic acid generated in the fermentation of MG1655(DE3)∆*pfkA*∆*pfkB*∆*zwf-*pKGGY was re-assimilated at the end of fermentation caused by the upregulated expression of the *acs* gene (Figure 6D,E). The acetate production of MG1655(DE3)∆*pfkA*∆*pfkB*∆*zwf-*pKGGY was reduced by more than 90% compared to that of MG1655(DE3)-pKGGY. These results indicated that blocking the EMP and PPP could promote acetate re-assimilation in *E. coli*, which further increased the substrate conversion efficiency of the mutant strain.

In conclusion, an *E. coli* platform strain with high F-6-P supply was constructed by blocking the EMP and PPP. Through introduction of glycerol consumption pathway and the GlcNAc biosynthesis pathway, the synergistic glucose and glycerol cofeeding strategy was successfully applied for GlcNAc production in this study. Ultimately, the fermentation medium was optimized in order to enhance the growth of the final mutant, resulting in a high GlcNAc yield of 0.64 mol/mol glucose. The mutant MG1655(DE3)∆*pfkA*∆*pfkB*∆*zwf* developed here is a promising host with minimal accumulation of acetate byproduct, which could be further engineered for other forms of valuable biochemical production.

## 4. Materials and Methods

### 4.1. Strains and Culture Media

All the strains used in this study are listed in Table 1. *E. coli* strain Top 10 was employed for DNA cloning. *E. coli* strain MG1655(DE3) was used as the parental strain for genetic engineering and GlcNAc production. *E. coli* strains were routinely cultured in Luria-Bertani (LB) medium supplemented with 100 μg/mL ampicillin (Amp) or 50 μg/mL kanamycin (Kan) when required. For characterization and fermentation of engineered strains, M9 minimal salt broths (M9s) (Na_2_PO_4_·7H_2_O 12.8 g/L, KH_2_PO_4_ 3.0 g/L, NaCl 0.5 g/L, NH_4_Cl 1.0 g/L, MgSO_4_·7H_2_O 0.5 g/L, CaCl_2_ 0.02 g/L) containing different carbon sources (10 g/L glucose or 5 g/L glycerol) were used. To increase the cell growth, various concentrations of pyruvate (0.1, 1 or 10 g/L), citric acid (0.1, 1 or 10 g/L) and LB broth (0.1%, 1%, 10%, 100%) were added to the medium.

### 4.2. Plasmid Construction

All the primers and plasmids used in this study are listed in Supplementary Table S1 and Table 1, respectively. For the construction of plasmid pRed_Cas9_recA, the gRNA expression cassette and homologous arms for *poxb* gene deletion on the plasmid pRed_Cas9_recA_∆*poxb*300 (MolecularCloud plasmid# MC_0000001) were eliminated through the modular assembly method [21]. To construct CRISPR-Cas9-assisting donor plasmids (harboring gRNA expression cassette and homology arms for target gene deletion), pEASY-T3 vector (TransGen, Beijing, China), which is a high copy-number plasmid, was chosen as the mother vector. For the attempts to delete the *pfkA*, *pfkB* and *zwf* genes in *E. coli* MG1655(DE3), the J23119 promoter fused with a 20-nt guiding sequence (*pfkA*: GTGTCTGACATGATCAACCG, *pfkB*: CACGTACATGTGGAAGCAAG, *zwf*: GCGTGCTGACTGGGATAAAG) was integrated into pEASY-T3 via TA cloning, and then the homology arms (~500 bp each) were inserted into the *Sbf*I and *Nde*I sites, generating plasmids p∆*pfkA*, p∆*pfkB* and p∆*zwf*, respectively.

For glycerol utilization and GlcNAc production, *glpK* from *P. pastoris* GS115, glmS* (glmS*72, encoding a mutated form of glucosamine-6-phosphate synthase) from *E. coli* [29], gna1* (*Ce*GAN1-Q155V/C158G, encoding a mutated form of glucosamine-6-phosphate *N*-acetyltransferase) from *C. elegans* [30] and *yqaB* from *E. coli* [17] were expressed under the native *pgi* promoter and cloned into the pEASY-T3 vector by TA cloning, creating plasmid pKGGY.

### 4.3. Mutant Screening

Genome editing in *E. coli* MG1655(DE3) was performed following the procedure described previously with some modifications [21,36]. In brief, plasmid pRed_Cas9_recA was transformed into *E. coli* MG1655(DE3) by electroporation, followed by plating on LB+Kan plates and culturing at 30 °C. For genome editing, the donor plasmid containing gRNA and homology arms was then transformed into MG1655(DE3)-pRed_Cas9_recA competent cells. The resulting cells were spread onto LB+Kan+Amp plates and incubated overnight at 30 °C. The transformant colonies were picked and inoculated in a LB+Kan+Amp liquid medium. The obtained cultures were then diluted serially and plated onto LB+Kan+Amp plates supplemented with 2 g/L D-arabinose to induce the expression of Cas9 nuclease and the λ-Red system. After the colonies were observed, the putative mutants were screened by colony PCR and then confirmed by Sanger sequencing (Supplementary Figures S1 and S2). To cure the pRed_Cas9_recA and donor plasmid in the newly obtained mutant, successive transferring at 37 °C was performed in an LB liquid medium without antibiotics. After five transfers, the culture was then diluted serially and spread onto LB plates for colony development. The pure mutant was obtained through colony PCR and then further verified by Kan and Amp selection.

### 4.4. Batch Fermentation

Batch fermentation with various GlcNAc-producing strains was carried out in a 1 L shake flask with 100 mL reaction volume at 37 °C and 200 rpm. M9s supplemented with different carbon sources (glucose and/or glycerol) and growth factors (pyruvate, citric acid or LB broth) were used as the fermentation medium. The strains were initially cultured in an LB medium to generate the seed culture (OD600 reached about 1.5). Then, a 2% inoculum of seed culture was used for all batch fermentations. Samples were taken every 6 h for the analysis. All fermentations were performed in triplicate.

### 4.5. Analytical Methods

Cell density was measured using a microplate reader at 600 nm (OD_600_). The glucose, glycerol and acetate concentrations in the fermentation broth were quantified using a high-performance liquid chromatography system (LC-20A, Shimadzu, Kyoto, Japan) equipped with a Sugar-ParkI column (Waters, Milford, MA, USA) and refractive index detector (RID). The mobile phase was ddH_2_O at a flow rate of 0.6 mL/min at 80 °C. The GlcNAc concentration was determined by an LC-MS system (Sciex TripleTOF 6600 interfaced with the UHPLC Agilent 1290 Infinity I) equipped with an ACQUITY UPLC BEH Amide column (21 mm × 100 mm, 1.7 µm). The mobile phases were a blend of solvent A (0.1% formic acid in water) and solvent B (0.1% formic acid in acetonitrile) at a flow rate of 300 µL/min at 45 °C.

### 4.6. RT-qPCR Analysis

The primers for RT-qPCR are shown in Supplementary Table S1. Cells cultivated in various media were harvested during the exponential growth phase (OD600 reached about 1.5). Total RNA extraction (RNA-easy Isolation Reagent, Vazyme, Beijing, China) and the reverse transcription of cDNA (HiScript III RT SuperMix for qPCR, Vazyme, Beijing, China) were conducted according to the manufacturer’s instructions. RT-qPCR was carried out using a QuantStudio 6 Flex system (Applied Biosystems, Foster City, CA, USA) and ChamQ Universal SYBR qPCR Master Mix (Vazyme, Beijing, China). RT-qPCR were performed following the procedure described by Lu et al. [37]. The transcription levels of the target genes were analyzed by the 2^−^^∆∆Ct^ method [38], where the 16S rRNA gene was used as the internal standard. Each sample was run in triplicate.

## Figures and Tables

**Table 1:** Bacterial strains and plasmids used in this study.

Strains/Plasmids	Relevant Characteristics	Sources
Strains		
*E. coli*		
Top 10	*F^−^, mcrA*, *Δ(mrr-hsdRMS-mcrBC)*, *φ80*, *lacZΔM15*, *ΔlacX74*, *nupG*, *recA1*, *araD139*, *Δ(ara-leu)7697*, *galE15*, *galK16*, *rpsL(StrR)*, *endA1*, *λ^−^*	Invitrogen
MG1655(DE3)	K-12 *F^–^*, *λ*(DE3), *ilvG^–^*, *rfb-50*, *rph-1*, wild-type strain	[35]
MG1655(DE3)∆*pfkA*	MG1655(DE3), *pfkA* deletion mutant	This work
MG1655(DE3)∆*pfkB*	MG1655(DE3), *pfkB* deletion mutant	This work
MG1655(DE3)∆*pfkA*∆*pfkB*	MG1655(DE3), *pfkA* and *pfkB* double-deletion mutant	This work
MG1655(DE3)∆*zwf*	MG1655(DE3), *zwf* deletion mutant	This work
MG1655(DE3)∆*pfkA*∆*pfkB*∆*zwf*	MG1655(DE3), *pfkA*, *pfkB* and *zwf* triple-deletion mutant	This work
MG1655(DE3)-pKGGY	MG1655(DE3), harboring plasmid pKGGY	This work
MG1655(DE3)∆*pfkA*∆*pfkB-*pKGGY	MG1655(DE3)∆*pfkA*∆*pfkB*, harboring plasmid pKGGY	This work
MG1655(DE3)∆*pfkA*∆*pfkB*∆*zwf-*pKGGY	MG1655(DE3)∆*pfkA*∆*pfkB*∆*zwf*, harboring plasmid pKGGY	This work
Plasmids		
pRed_Cas9_recA_∆*poxb*300	Exo, bet, gam, *recA*, arabinose operon, Cas9, gRNA and homologous arms for *poxb* deletion	[21]
pRed_Cas9_recA	Derived from pRed_Cas9_recA_∆*poxb*300, Exo, bet, gam, *recA*, arabinose operon and Cas9	This work
p∆*pfkA*	pEASY-T3, gRNA and homologous arms for *pfkA* deletion	This work
p∆*pfkB*	pEASY-T3, gRNA and homologous arms for *pfkB* deletion	This work
p∆*zwf*	pEASY-T3, gRNA and homologous arms for *zwf* deletion	This work
pKGGY	pEASY-T3, harboring *glpK*, glmS* (glmS*72, a mutated form of glucosamine-6-phosphate synthase), gna1* (CeGAN1-Q155V/C158G, a mutated form of glucosamine-6-phosphate N-acetyltransferase) and *yqaB* expression cassettes	This work

## Data Availability

Data is contained within the article or Supplementary Materials.

## Supplementary Materials

The following supporting information can be downloaded at: https://www.mdpi.com/article/10.3390/ijms23020773/s1.
