# Supplementary material for: Synergetic Fermentation of Glucose and Glycerol for High-Yield N-Acetylglucosamine Production in Escherichia coli
Source: Int J Mol Sci. 2022 Jan 11;23(2):773. doi: 10.3390/ijms23020773 (PMC8775389; doi:10.3390/ijms23020773)
Supplement: Supplementary file 1 [file ijms-23-00773-s001.zip › ijms-1446912-supplementary.pdf]

Supplementary Materials:

Synergetic fermentation of glucose and glycerol for high yield N-acetylglucosamine production in *Escherichia coli*

Kaikai Wang#, Xiaolu Wang#, Huiying Luo, Yaru Wang, Yuan Wang, Tao Tu, Xing Qin, Yingguo Bai, Huoqing Huang, Bin Yao, Xiaoyun Su*, Jie Zhang*

State Key Laboratory of Animal Nutrition, Institute of Animal Science, Chinese Academy of Agricultural Sciences, Beijing 100193, China

# Kaikai Wang and Xiaolu Wang contributed equally to this work.

* Corresponding author:

Xiaoyun Su

No.2 Yuanmingyuan West Road, Haidian district

Beijing, 100193 China

Tel: +86-10-62599910

E-mail: suxiaoyun@caas.cn

Jie Zhang

No.2 Yuanmingyuan West Road, Haidian district

Beijing, 100193 China

Tel: +86-10-62599910

E-mail: zhangjie09@caas.cn

Supplementary Table S1. Primers used in this study.

***pfkA*, *pfkB* and *zwf* deletion using CRISPR-Cas9 system**		
Genes	Primers	Sequences （5′→3′）
pfkA-gRNA	pfkA-gRNA-F	TTGACAGCTAGCTCAGTCCTAGGTATAATGCTAGCGTGTCTGACATGATCAACCGGTTTTAGAGCTAGAAATAGCAAG
	gRNA-R	TCGGATCCACTAGTAACCATC
pfkA-homologous arms	pfkA-N-F	AATCACTAGTGAATTCGCGGCCGTCGGCATCTATATTTTATATAGCG
	pfkA-N-R	GAAATCAGACTACCTCTGAACTTTGGAATGCAAAATGAAATCTGTTGC
	pfkA-C-F	TCCAAAGTTCAGAGGTAGTCTGATTTCGGAAAAAGGCAGATTC
	pfkA-C-R	ATGCATCCAACGCGTTGGGAGCTCTCCCACCGTGTGACTGACGAATC
Mutant detection	A-JC-F	TTATCCTTGTCTCGTTTCAGCGTTGGGTGGTGCG
	A-JC-R	GTTTGATCCACTCTTTATCAATC
pfkB-gRNA	pfkB-gRNA-F	TTGACAGCTAGCTCAGTCCTAGGTATAATGCTAGCCACGTACATGTGGAAGCAAGGTTTTAGAGCTAGAAATAGCAAG
pfkB-homologous arms	pfkB-N-F	CAATCACTAGTGAATTCGCGGCCGCAGCGACCAGGCAGTGGTGTGTC
	pfkB-N-R	GGGGAATGTTTTTGCATTTCCTCCTATAGGCTGA
	pfkB-C-F	GGAGGAAATGCAAAAACATTCCCCCAGCATTGGGGGAATCATCAC
	pfkB-C-R	TGCATCCAACGCGTTGGGAGCTCTCCTCAAAAGACGACTGATTGCCTGC
Mutant detection	B-JC-F	CATCGCGCTCTCGATAGCCGTTAT
	B-JC-R	GCGCAAATGCCATGCGGCATGGA
zwf-gRNA	zwf-gRNA-F	TTGACAGCTAGCTCAGTCCTAGGTATAATGCTAGCGCGTGCTGACTGGGATAAAGGTTTTAGAGCTAGAAATAGCAAG
zwf-homologous arms	zwf-N-F	AATCACTAGTGAATTCGCGGCCGCGTGTCCATGCTGCGACAGAAACG
	zwf-N-R	GCAGATAGTCATTCTCCTTAAGTTAACTAACCCGGTACTTAAGC
	zwf-C-F	AGTTAACTTAAGGAGAATGACTATCTGCGCTTATCCTTTATGG
	zwf-C-R	TCCAACGCGTTGGGAGCTCTCCCGCATTCGCTTCATGCAGGGCTTTAC
Mutant detection	zwf-JC-F	ACGGCACAAACACCGCAGGC
	zwf-JC-R	ACATGATCAAGCGTTGCCATTGC

**Plasmid pKGGY construction**		
Genes	Primers	Sequences （5′→3′）
pgi promotor-1	Ppgi-F1	TTCTGGTGACAACCCAGGGGATTCAGCCCCTG
	Ppgi-R1	AGTGGTGTATAGTCTTTTCCCATTAGCAATACTCTTCTGATTTTGAG
glpk	pichi-glpk-F	CTTTAAGAAGGAGATATACCATGGGAAAAGACTATACACCAC
	pichi-glpk-R	CGTCGCATCAGGCATAAAGCAGATTAAGCAGTGTCCTTAAGCCAGCCC

pgi promotor-2	pgi-F2	GCAAAGGGCTGGCTTAAGGACACTGCTTAAATCACCTCGGCGATGCACCCC
	Ppgi-R2	TCGCGCCAACAATTCCACACATTAGCAATACTCTTCTGATTTTGAG
glms	Glms-F	GAGTATTGCTAATGTGTGGAATTGTTGGCGCGATCGCGCAACGTGATG
	Glms-R	CTGATCTAGATTTCTCCATAGAAGCTTTCGTCTACCTCGAGTTACTCAACCGTAACCGATTTTGCC
pgi promotor-3	Ppgi-F3	GCAAAATCGGTTACGGTTGAGTAACCTCAGGTGTTATCACAGGACTGGC
	Ppgi-R3	CTCGCATCAAAAATATGGCTCATTAGCAATACTCTTCTGATTTTGAGA
gna-1	gna1-F	CTCAAAATCAGAAGAGTATTGCTAATGAGCCATATTTTTGATGCGAGCG
	gna1-R1	GTAACGGTGTTGGAGTTAAAAGCGCTGGGTCATAAAGTTGCCATC
pgi promotor-4	Ppgi-F4	AACTTTATGACCCAGCGCTTTTAACTCCAACACCGTTACTTGGGC
	Ppgi-R4	AACCTGCATAACGCTCGTACATTAGCAATACTCTTCTGATTTTGAG
yqaB	yqaB-F	GAGTATTGCTAATGTACGAGCGTTATGCAGGTTTAATTTTTGA
	yqaB-R	CATGCTGATCTAGATTTCTCCATAGTCACAGCAAGCGAACATCCACGG
16s terminator	16stt-F	TCGAGGTAGACGAAAGCTTCTATGGAGAAATCTAGATCAGCATGATGTC
	16stt-R	CACCGTATTGTCCGCTTAATTATTAGTC

Primers used for RT-qPCR

Genes	Primers	Sequences （5′→3′）
16s	16s-F	CGGTGAATACGTTCCCGG
	16s-R	GGTTACCTTGTTACGACTT
fbaA	fbaA-F	TCTTGTTCGGCTGATCTTCG
	fbaA-R	ATACCGATACCCAATGGG
fbaB	fbaB-F	CCGTCAGCCATCGATTTCTT
	fbaB-R	CTGGGTTGATAAACTCCG
zwf	zwf-F	CAGAACATTTGGTCGGCAGA
	zwf-R	AAAGTGCCGGGATATCTGGAAG
pgi	pgi-F	AAGTCGATGTTCTCTGGCGA
	pgi-R	GGTTTTCATCTTCTCCAGCACC
pgl	pgl-F	CTGAGAGCCAGCAAATTCAC
	pgl-R	TCAGGGCGAACACCAACATAGA
fruA	fruA-F	GTACGCTGCTTTGTTTACCGGA
	fruA-R	TGGCGATGATCTACCTGATC
fruK	fruK-F	ACCTGCTACTAACGCTTCAC

	fruK-R	GCTCGGTCAGTTCGATAT
acs	ACS-F	ACCAGTCGTGAGTTGGAATC
	ACS-R	TGTGGTGGCGATTTATATGCCG

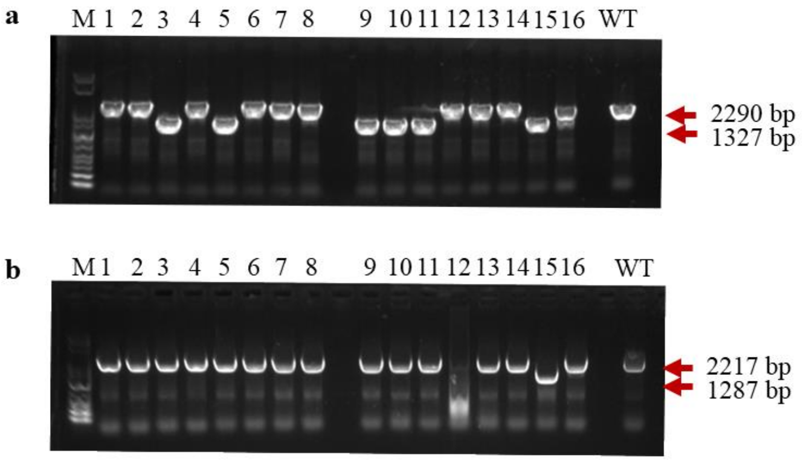

Supplementary Figure S1. *pfkA* (a) and *pfkB* (b) genes knockout in *E. coli* MG1655(DE3). For *pfkA* knockout detection, the PCR product sizes of wild-type strain and Δ*pfkA* mutant are 2290 bp and 1327 bp, respectively. For *pfkB* knockout detection, the PCR product sizes of wild-type strain and Δ*pfkB* mutant are 2217 bp and 1287 bp, respectively. M, 1-kb DNA ladder; WT, genomic DNA of wild type *E. coli* MG1655(DE3) as the control.

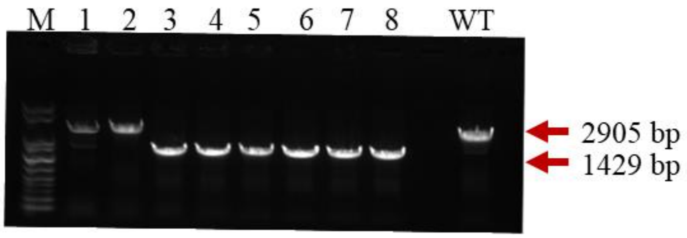

Supplementary Figure S2. *zwf* gene knockout in mutant MG1655(DE3)Δ*pfkA*Δ*pfkB*. The PCR product sizes of MG1655(DE3)Δ*pfkA*Δ*pfkB* and MG1655(DE3)Δ*pfkA*Δ*pfkB*Δ*zwf* are 2905 bp and 1429 bp, respectively.
